# Proteome profiling of secreted and membrane vesicle associated proteins of an invasive and a commensal *Staphylococcus haemolyticus* isolate

**DOI:** 10.1016/j.dib.2018.11.147

**Published:** 2019-01-11

**Authors:** Jorunn Pauline Cavanagh, Fatemeh Askarian, Maria Pain, Jack-Ansgar Bruun, Ilona Urbarova, Sun Nyunt Wai, Frank Schmidt, Mona Johannessen

**Affiliations:** a Department of Paediatrics, University Hospital of North Norway, Tromsø, Norway; b Paediatric Research Group, Department of Clinical Medicine, Faculty of Health Sciences, UiT - The Arctic University of Norway, Tromsø, Norway; c Research Group of Host Microbe interaction, Department of Medical Biology, UiT- The Arctic University of Norway, Tromsø, Norway; d Faculty of Chemistry, Biotechnology and Food Science, Norwegian University of Life Sciences (NMBU), 1432 Ås, Norway; e Proteomics Platform Facility, Department of Medical Biology, UiT - The Arctic University of Norway, Tromsø, Norway; f Department of Molecular Biology, Umeå University, Sweden; g Interfaculty Institute for Genetics and Functional Genomics, University Medicine Greifswald, Greifswald, Germany; h Proteomics Core, Weill Cornell Medicine-Qatar, Education City, PO 24144, Doha, Qatar

## Abstract

Bacterial membrane vesicles (MVs) mediate bacterial virulence by enabling secretion and long distance delivery of bacterial effector molecules. *Staphylococcus haemolyticus* has now been demonstrated to produce membrane vesicles (MVs). The protein content of *S. haemolyticus* MVs was identified by Mass spectrometry and compared to proteins identified in the total secretome. This information is presented in this data article. Further background and interpretation of the data can be found in the article: Comparative exoproteome profiling of an invasive and a commensal *S. haemolyticus* isolate (Cavanagh et al., in press). Data are available via Proteome Xchange with identifier PXD010389.

**Specifications table**

**Table:** 

Subject area	*Microbiology and Molecular Biology*
More specific subject area	*Protein content of bacterial membrane vesicles compared to total secretome*
Type of data	*Tables*
How data was acquired	*Mass spectroscopy, Thermo Fisher Scientific EASY-nLC1000 system*
Data format	*Analyzed*
Experimental factors	*Protein samples were reduced and alkylated with dithiothreitol and iodoacetoamide, prior to digestion with a 1:20 ratio of trypsin.*
Experimental features	*Bacterial membrane vesicles was harvested from overnight cultures, and presence was confirmed by electron microscopy before purification. Proteins both from membrane vesicles and the total Secretome were the precipitated and identified by mass spectrometry.*
Data source location	*Tromsø, Norway*
Data accessibility	*Data are available via Proteome Xchange with identifier*PXD010389

**Value of the data**

• These data provide a comparative analysis of secreted proteins identified in the total secretome and in the membrane vesicle cargo of a commensal and a clinical strain.
• An enrichment analysis was performed, comparing the proteins found in the membrane vesicle samples, to the proteins found in the total secretome.
• These data would be valuable in further studies comparing secreted and membrane vesicle (MV) associated proteins found in other Staphylococcus species.
• These data are valuable in further comparative analyses of membrane vesicle cargo between *S. haemolyticus* and other gram positive species.

## 1 Data

*Staphylococcus haemolyticus* is a skin commensal now emerging as an opportunistic pathogen, apart from being multiresistant to several antimicrobial agents, little is known about its virulence factors [2]. Bacterial membrane vesicles (MVS) are mediators of bacterial virulence, and has recently been found in gram positive bacteria [3]. It has been shown that *S. haemolyticus* produces MVs, and that the protein cargo is strain specific [1]. The data presented in this article provide information on the proteins identified in the MV cargo and in the total secretome of a clinical and a commensal *S. haemolyticus* strain. Unique and common proteins found in MVs and the total Secretome of both strains are presented. Proteins found to be enriched in the MV cargo as compared to the total Secretome are presented for both strains.

## 2 Experimental design, materials and methods

**Fig. 1:**
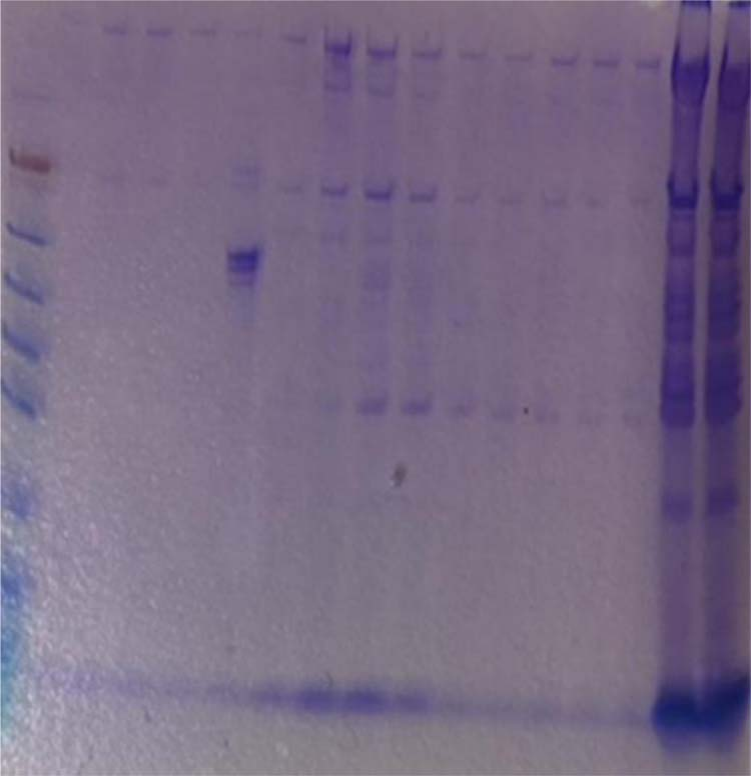
Presence of proteins in the different fractions after OptiPrep were visualized by SDS PAGE Comassie Blue staining.

Membrane vesicles (MVS) were isolated from a commensal *S. haemolyticus* strain (57-1), and a clinical *S.haemolyticus* strain (51-08), according to the methods described in [1], [4]. Briefly, proteins secreted into the bacterial growth medium was harvested after the culture was centrifuged and filtered through a 0.22 µm polyethersulfone membrane (Millipore express plus, Merck Millipore, Burlington, USA). MVs were isolated by ultra centrifugation and purified using an Optiprep gradient, presence of proteins in the different OptiPrep fractions were visualized by SDS PAGE Comassie Blue staining, Fig. 1.

Proteins in the MV samples and the total secretome were precipitated and digested in solution, prior to protein identification by Mass spectrometry using a Thermo Scientific Q-Exactive mass spectrometer.

The raw data were processed in the MaxQuant software v1.6.0.16 using label-free intensity based absolute quantification (iBAQ) according to the method published in [1]. The raw data are deposited in the Proteome Xchange with identifier PXD010389.

A quantitative comparison of proteins secreted by the two bacterial strains was performed using the relative iBAQ values (riBAQ) in Perseus programme v1.5.6.0 [5]. Proteins with minimum two peptides identified was used. All contaminants were first filtered out and the relative iBAQ values for each sample were log10 transformed. Missing values were replaced from normal distribution using width = 0.3 and downshift = 1.8 settings.

**Fig. 2:**
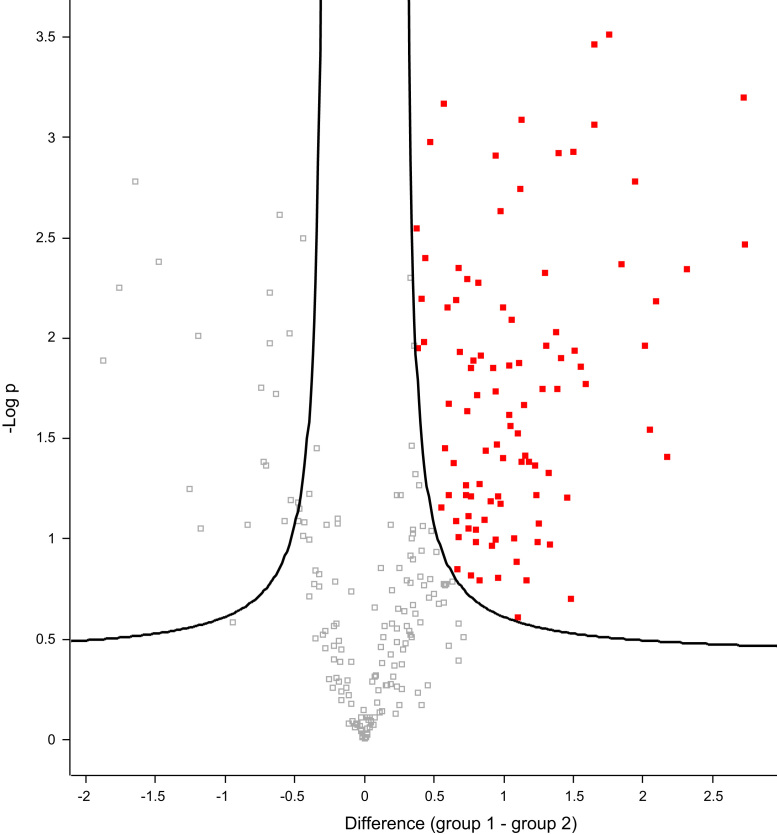
Volcano plot used to visualize the differentially secreted proteins in the MVs and in the total secretome of strain 51-08, enriched proteins are depicted as red filled squares.

**Fig. 3:**
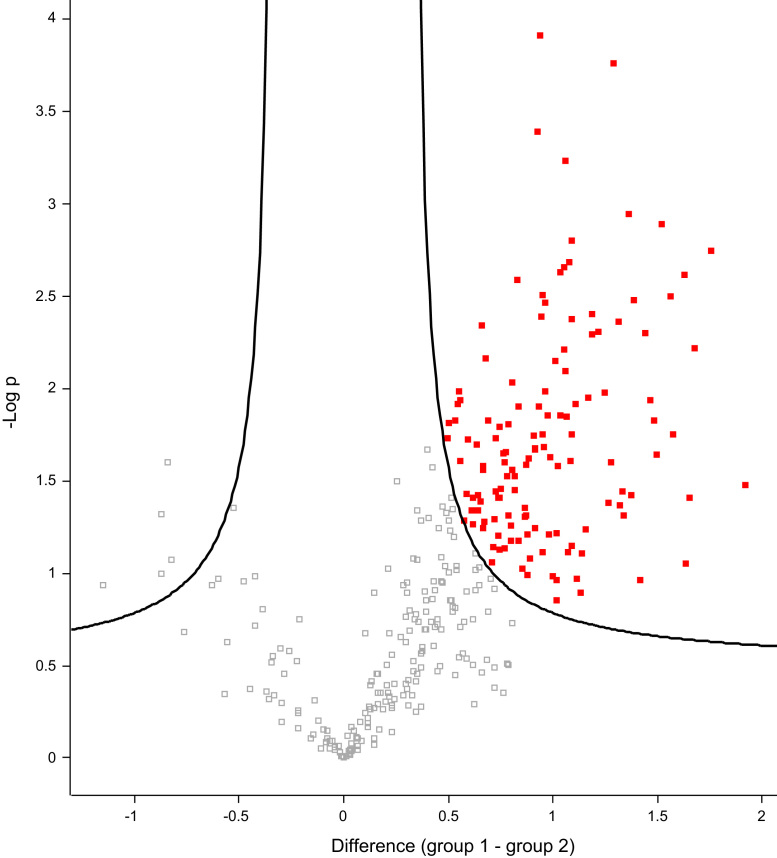
Volcano plot used to visualize the differentially secreted proteins in the MVs and in the total secretome of strain 57-1, enriched proteins are depicted as red filled squares.

Differentially secreted proteins in the MV cargo and in the total secretome of strains 57-1 and 51-08, were then visualized using Volcano plot with FDR < 0.05 and artificial within group variance s0 = 0.3, Fig. 2, Fig. 3. For qualitative comparisons, only proteins present in at least two replicates in each group were considered further. The rIBAQ values for proteins identified in the total secretome and in the MV cargo of the commensal and the clinical strain respectively is presented in Tables S1 and S2.

Functional annotation and grouping of proteins into orthologous groups were performed using EggNOG version 4.5.1 [6], while the cellular localisation of each protein was predicted using the PSORTb subcellular localisation tool version 3.0.2 [7]. The presence of potential signal sequences in each peptide was identified using SignalP v4.1 [8], [9]. Secretome P v2.0 was used to predict non-classical protein secretion [10].

Proteins uniquely found in the MV cargo of the commensal and clinical strain are presented in Tables S3 and S4 respectively, while common proteins found in the MV cargo in both strains are presented in Table S5.

It has previously been shown that MVs are enriched in virulence factors [11]. An enrichment analysis was performed comparing the MV cargo to the total secretome. If proteins were detected with a threshold detection rate of FDR 0.05 in the MV sample as compared to in the TS, these proteins were defined as enriched.

Proteins enriched in the MVs compared to the total secretome of the commensal strain and the clinical strain are shown as red squares in the volcano plots in Fig. 2, Fig. 3. Enriched proteins are listed in Tables S6 and S7.

Proteins uniquely found in the total secretome of the commensal and clinical strain are presented in Tables S8 and S9 respectively, while common proteins found in the total secretome of both strains are presented in Table S10.
